# MLH1 enhances the sensitivity of human endometrial carcinoma cells to cisplatin by activating the MLH1/c-Abl apoptosis signaling pathway

**DOI:** 10.1186/s12885-018-5218-4

**Published:** 2018-12-29

**Authors:** Yue Li, Shihong Zhang, Yuanjian Wang, Jin Peng, Fang Fang, Xingsheng Yang

**Affiliations:** 1 Department of Obstetrics and Gynecology, Qilu Hospital of Shandong University, No. 107, Wenhua Xi Road, Jinan, Shandong 250012 People’s Republic of China; 2 Department of Obstetrics and Gynecology, Weihai Municipal Hospital, No. 70, Heping Road, WeiHai, Shandong 264200 People’s Republic of China; 3 Huaxi Clinical Medical College of Sichuan University, Jiang’an campus, Chengdu City, Sichuan 610207 People’s Republic of China

**Keywords:** Endometrial carcinoma, siRNA-MLH1, ADV-MLH1, Cisplatin sensitivity, Individualized therapy

## Abstract

**Background:**

MLH1 plays a critical role in maintaining the fidelity of DNA replication, and defects in human MLH1 have been reported. However, the role of MLH1 in endometrial carcinoma has not been fully investigated. Therefore, we aimed to study the role of MLH1 in the sensitivity of human endometrial carcinoma cells to cisplatin.

**Methods:**

In this study, we detected the expression of MLH1 in Ishikawa and RL95–2 cells. MLH1-siRNA and ADV-MLH1 were adopted for the silencing and overexpression of MLH1, respectively. Real-time polymerase chain reaction, Western blotting, cell proliferation assays, and cell cycle and apoptotic analyses by flow cytometry were employed to explore the underlying mechanism. A mouse xenograft model was used to investigate the effect of MLH1 on tumor growth after treatment with cisplatin.

**Results:**

Over-expression of MLH1 in Ishikawa cells dramatically increased the sensitivity of cells to cisplatin and enhanced cell apoptosis. By contrast, knockdown of MLH1 yielded the opposite effects in vitro. Mechanistically, cisplatin induced the MLH1/c-Abl apoptosis signaling pathway in ADV-MLH1-infected endometrial carcinoma cells, and these effects involved c-Abl, caspase-9, caspase-3 and PARP. Altogether, our results indicate that ADV-MLH1 might attenuate Ishikawa cell growth in vivo, resulting in increased cisplatin sensitivity.

**Conclusions:**

MLH1 may render endometrial carcinoma cells more sensitive to cisplatin by activating the MLH1/c-Abl apoptosis signaling pathway. In addition, an applicable adenovirus vector (ADV-MLH1) for MLH1 overexpression in endometrial carcinoma was generated. Thus, ADV-MLH1 might be a novel potential therapeutic target for endometrial carcinoma.

**Electronic supplementary material:**

The online version of this article (10.1186/s12885-018-5218-4) contains supplementary material, which is available to authorized users.

## Background

Endometrial carcinoma is a common malignant tumor in the female reproductive system. Women with hereditary nonpolyposis colorectal cancer exhibit a 40 to 60% cumulative lifetime risk for endometrial carcinoma, which arises as a result of a genetic predisposition to the disease, characterized by mutations in DNA mismatch repair (MMR) genes such as *MLH1* and *MSH2* [1]. Defects in MMR proteins give rise to genome instability, which is a characteristic of most cancers, especially hereditary cancers [2, 3]. Loss of DNA mismatch repair caused by MMR deficiency also accounts for the cytotoxicity induced by specific types of DNA-damaging chemotherapeutic agents (e.g., alkylating agents and cisplatin) [4, 5]. Thus, MMR is essential for effective cancer therapy and individual health.

Various models have demonstrated drug resistance caused by low levels of the MLH1 protein in ovarian and esophageal tumor samples following cisplatin (cis-dichlorodiammine platinum, CDDP)-based chemotherapy. Additionally, several studies, which examined MMR protein levels and microsatellite instability in germ cell tumors from patients receiving cisplatin-based chemotherapy, have shown the prognostic value of prechemotherapy MMR protein status in these tumors [6, 7]. Sawant et al. demonstrated that loss of base excision repair and MMR proteins gives rise to cisplatin resistance, and these two pathways share the same mechanism in mediating cisplatin sensitivity [8, 9]. It has also been observed that decreased cellular cytotoxicity is induced by increased repair of cisplatin interstrand crosslinks in the absence of MMR proteins [10]. The potential relevance of these findings underscores the need for a greater understanding of the role of MLH1 in mediating cisplatin sensitivity. In this study, we investigated the role of MLH1 in the sensitivity of human endometrial carcinoma cells to cisplatin and generated an adenovirus vector (ADV) ADV-MLH1 that can be widely applied for selective overexpression of MLH1, which represents a potential therapeutic target for endometrial carcinoma. No similar research has been reported internationally.

## Methods

### Cell culture

Ishikawa and RL95–2 cells were generously donated by the Gynecologic Oncology Laboratory at Qilu Hospital in Shandong Province, China. RL95–2 cells were maintained in Dulbecco’s modified Eagle’s medium/F-12 media (HyClone, Biological Industries, Israel) with 10% fetal bovine serum (FBS, Invitrogen, USA) with antibiotics, whereas Ishikawa cells were maintained in Roswell Park Memorial Institute (RPMI) modified medium (HyClone, Biological Industries, Israel) supplemented with 10% FBS (Invitrogen, USA) with antibiotics. All cell lines were cultured in a humidified atmosphere of 5% CO_2_ at 37 °C. Half of the medium was replaced with fresh medium at 3-day intervals until the attached cells reached 70–80% confluence in our experiments.

All experimental procedures were approved by the Laboratory Animal Ethics Committee of Qilu Hospital, Shandong University. The principles outlined in the ARRIVE (Animal Research: Reporting of In Vivo Experiments) guidelines and the Basel declaration (including the 3 R concept) were considered when planning experiments.

### Reagents and antibodies

Cisplatin was purchased from Sigma-Aldrich (USA), dissolved in dimethyl sulfoxide (DMSO, Solarbio, Beijing, China) to a stock concentration of 10 mM, and stored in single-use aliquots at − 80 °C. An anti-MLH1 antibody was purchased from Abcam (ab92312, United Kingdom). Anti-p-c-Abl, anti-cleaved caspase-3, anti-cleaved caspase-9, and anti-cleaved PARP antibodies were purchased from Cell Signaling Technology Inc. (China). Anti-BCL-2 antibody was purchased from Proteintech Group Inc. (USA). An anti-β-actin antibody was purchased from Zhongshan Jinqiao biotechnology Co., Ltd. (Beijing, China).

### Real-time polymerase chain reaction (PCR) for measurement of MLH1 transcript levels

Total RNA was isolated using TRIzol (Invitrogen, USA) according to the manufacturer’s instructions. First-strand cDNA synthesis was performed using the Moloney murine leukemia virus (M-MLV) reverse transcriptase enzyme (Invitrogen, USA) according to the manufacturer’s protocol. Transcript levels were quantified using SYBR Premix Ex Taq (Takara Bio Inc., Japan) in an Applied Biosystems StepOne Plus Real-time PCR System. Detailed methods are available for download at www.takara-bio.com. U6 served as an endogenous control. The fold-changes in mRNA expression levels were determined using the 2^−△△CT^ method [11, 12]. Primers were designed by Genepharma Company (Shanghai, China). The primer sequences are presented in Additional file 4: Table S1.

### Western blot analysis

Proteins were extracted after treatment using a standard method, and protein concentrations were determined using the Bradford method according to Kruger [13]. Proteins were extracted with RIPA buffer (50 mM Tris pH 8.0, 150 mM NaCl, 1% NP-40, 1 mM ethylenediaminetetraacetic acid (EDTA), and 0.1% sodium dodecyl sulfate (SDS) containing protease inhibitors). Total protein (30 μg) was separated in a 12% SDS gel and transferred to Immobilon-P transfer membranes (Merck Millipore, Ltd. Tullagreen, Carrigtwohill, Co. Cork IRL Rev. Size: 0.2 μm) after electrophoresis. After blocking with 5% nonfat dry milk, the membranes were probed with primary antibodies recognizing human MLH1 (1:1000), p-c-Abl (1:1000), cleaved caspase-3 (1:800), cleaved caspase-9 (1:500), cleaved PARP (1:500), and Bcl-2 (1:1000), followed by a horseradish peroxidase-linked secondary antibody (goat anti-rabbit IgG, 1:1000). β-actin (1:1000) served as an endogenous control. Immunoreactive proteins were visualized using the Immobilon Western Chemiluminescent substrate (ECL; Millipore Corporation, Billerica, MA, USA). The density of all proteins relative to β-actin was quantified with ImageJ (National Institutes of Health, USA). The experiment was repeated in triplicate.

### MLH1 overexpression and silencing

The MLH1-pAD-kan adenovirus vector (ADV-MLH1) was utilized to upregulate MLH1 (Additional file 1: Figure S1, Additional file 2: Figure S2 and Additional file 3: Figure S3). ADV-NC served as a negative control (Additional file 4: Table S1). Cells were plated in 6-well plates and infected at 30 to 40% confluence using ADV-MLH1 (1 × 10^12^ vp/ml, Multiplicity of infection (MOI) = 200) mixed with serum-free medium. The medium was changed after 12 h. After treatment, cells were harvested at different time points and prepared for future use. We selected the optimal MLH1 siRNA fragment to silence MLH1. Cells were transfected with the indicated MLH1-siRNA (MLH1-homo-528/1832/2431) (synthesized by Genepharma, Shanghai) using the Endofectin-MAX Transfection Reagent (Genecopoeia, Rockville, USA) according to the manufacturer’s instructions. The sequences of MLH1-homo-528, MLH1-homo-1832 and MLH1-homo-2431 are presented in Additional file 4: Table S1. Briefly, the cells were plated in 6-well plates. At the time of transfection, cell density was maintained at 50 to 60%, and the medium was changed to complete medium at 6-h intervals. The cells were harvested at different time points for the detection of expression levels.

### Cell proliferation assays

An enhanced cell counting kit-8 (CCK-8, Beyotime, Beijing) assay was utilized to determine cell proliferation as previously described [14]^**.**^ Cells were plated in 96-well plates at a density of 8000 to 10,000 cells/well. Viable cell absorbance values were determined using a NanoQuant Infinite M200 PRP (TECAN, Austria) at 490 nm, and the data were analyzed with SoftMax software. Cell viability was expressed by normalizing untreated cells to 100%. The experiment was performed in triplicate for each cisplatin concentration.

### Flow cytometry analysis of the cell cycle and apoptosis

The cell cycle was analyzed using a Cell Cycle Detection Kit (BB-4104, BestBio, Shanghai) according to the manufacturer’s instructions. The cells were harvested by centrifugation and then fixed in 75% cold ethanol overnight after washing three times with PBS. Each sample was washed and resuspended in 500 μl of propidium iodide (PI) working solution for 30 to 60 min in the dark before detection using a FACSCalibur flow cytometer (Becton, Dickinson and Company, USA). The percentage of cells was further analyzed with ModFit LT software. Cell apoptosis was measured using an Annexin V-FITC/PI staining kit (BB-4101, BestBio, Shanghai) according to the manufacturer’s guidelines. The cells were counted and adjusted to 1 × 10^6^ cell/ml. Then**,** a 2 ml aliquot of the cells was inoculated into each well of a 6-well plate, and the cells were allowed to grow for 24 h prior to drug treatment. Flow cytometry was performed using a FACSCalibur flow cytometer (Becton, Dickinson and Company, USA) and processed using CellQuest Pro analysis software. The experimental methods used for flow-cytometric analysis in our study can be found in previous reports [15, 16].

### Animal experiments and immunohistochemistry staining

Six-week-old female BALB/c nude mice weighing 16 to 18 g were purchased from Vital River (Beijing, China). All animal experiments complied with the ARRIVE guidelines and were performed in accordance with the National Institutes of Health Guide for the Care and Use of Laboratory Animals (NIH Publications No. 8023, revised 1978). This experiment was designed for 6 animals per group. Briefly, 1 × 10^7^ Ishikawa cells were suspended in 200 μl of PBS and injected subcutaneously into the left flank of each mouse. When the tumor diameter reached 4 to 6 mm, the mice in each group received the first intratumoral injection of ADV-MLH1 and ADV-NC (each at 2 × 10^9^ VP/tumor). We repeated the intratumoral injection of the same adenoviral vector (2 × 10^9^ VP/tumor) at days 5, 9, 13 and 17. Cisplatin treatment was performed via intraperitoneal injection of 5 mg/kg cisplatin in 0.9% physiological saline at days 4, 11, 18 and 25. Tumor volume was calculated as follows: V (tumor) = D×(d^2^). (D = length, d = width, d < D).

MLH1 protein expression in mouse tumor tissue from each group (ADV-MLH1 + CDDP-treated mice, ADV-NC + CDDP-treated mice) was assessed by immunohistochemistry staining. Standard three-step, indirect immunohistochemical analysis was conducted in 4-μm tissue sections that had been transferred to glass slides and included the following steps: citrate antigen retrieval, endogenous peroxidase blockage and avidin-binding activity and di-aminobenzidine development [17]. Primary antibody against MLH1 (dilution, 1:200) (Abcam, ab92312, United Kingdom) was used, and the corresponding secondary antibodies were goat anti-rabbit IgG antibodies (cat. no. SP-9000; Zhongshan Jinqiao biotechnology Co., Ltd., Beijing, China). Staining patterns were analyzed by at least two experienced pathologists affiliated with the Department of Pathology of Qilu Hospital of Shandong University.

### Statistical analysis

All statistical analyses were performed in GraphPad Prism version 6.01. Differences between two or three groups were analyzed using Student’s t-test. *P*-values < 0.05 were considered statistically significant.

## Results

### MLH1 protein and mRNA levels in endometrial carcinoma cells subjected to different treatments

**Fig. 1:**
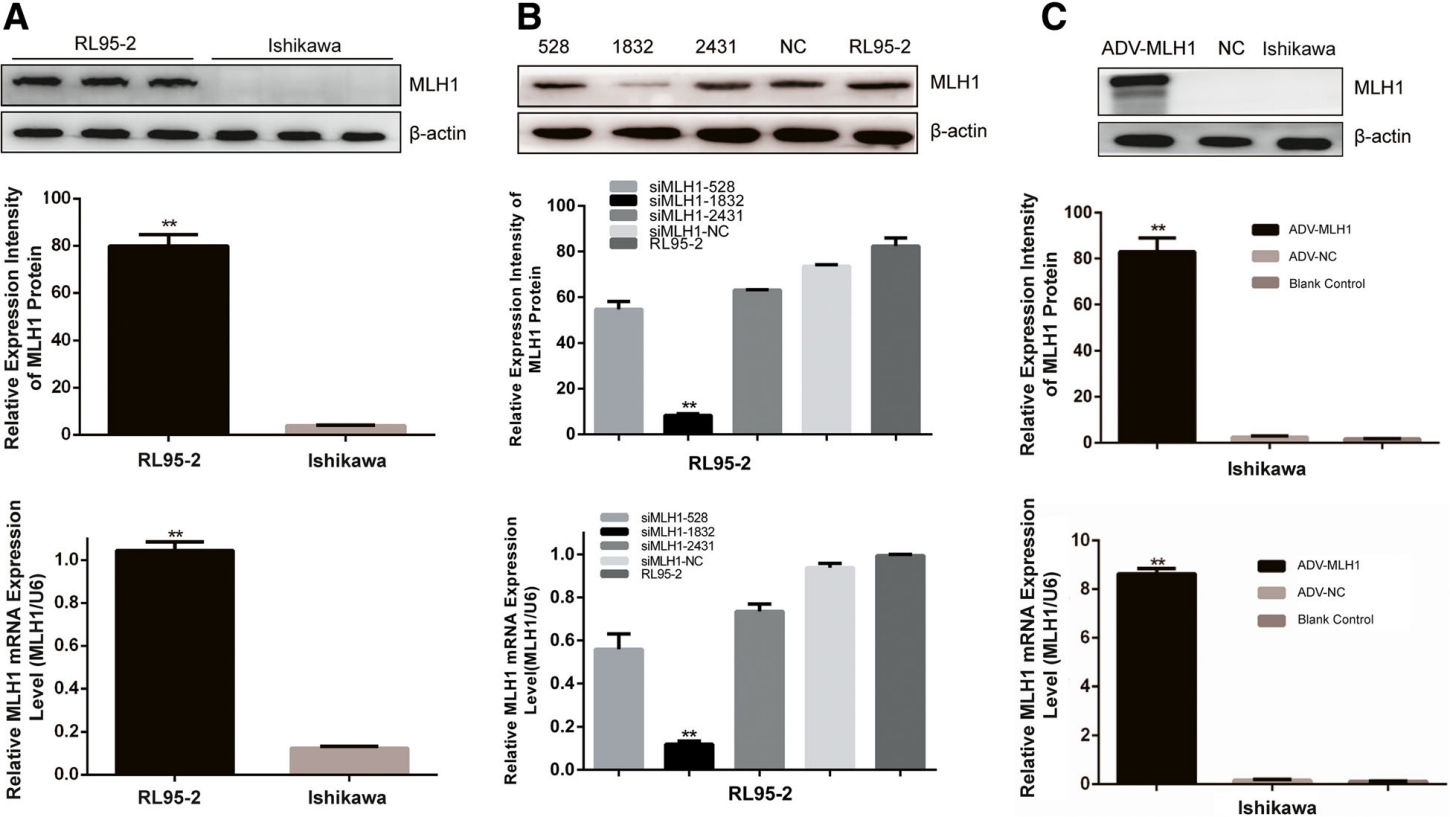
MLH1 protein and mRNA levels in cells subjected to different treatments. **a** MLH1 protein and mRNA expression levels in Ishikawa and RL95–2 cells were measured by Western blotting and qRT-PCR (**, *P* < 0.01). **b** Comparison of MLH1 protein and mRNA knockdown using siMLH1–528, siMLH1–1832 and siMLH1–2431 by Western blotting and qRT-PCR. si-MLH1-NC and untreated RL95–2 cells were used as controls. siMLH1–1832 dramatically downregulated MLH1 expression in RL95–2 cells compared with the siMLH1–528, siMLH1–2431 and siMLH1-NC groups (**, *P* < 0.01). **c** Western blotting and qRT-PCR showed upregulated MLH1 expression by ADV-MLH1 in Ishikawa cells (**, *P* < 0.01). β-actin was used as a loading control, and mRNA levels were normalized to U6. Data are presented as the mean ± SEM of three experiments

To study the role of MLH1 in endometrial carcinoma, we first confirmed the protein and mRNA levels of MLH1 in two human endometrial cancer cell lines (Ishikawa and RL95–2 cells) and investigated the overexpression and silencing of MLH1 in these cells. As shown in Fig. 1a, lower MLH1 expression was detected in Ishikawa cells than in RL95–2 cells (3.9% VS 79.9%; *P* < 0.01). Next, we analyzed MLH1 expression levels in cells subjected to different treatments. siMLH1–1832 reduced MLH1 protein levels by more than 90% in RL95–2 cells compared with siMLH1–528, siMLH1–243 and negative control siMLH1-NC. MLH1 silencing was confirmed by Western blotting and quantitative RT-PCR (qRT-PCR; *P* < 0.01; Fig. 1b). Additionally, we generated an adenovirus vector (ADV-MLH1) for overexpressing MLH1. Elevated MLH1 protein and mRNA levels in Ishikawa cells were confirmed by Western blotting and qRT-PCR compared with the controls (**, *P* < 0.01; Fig. 1c).

### MLH1 affected the sensitivity and cell cycle of endometrial carcinoma cells in response to cisplatin in vitro

**Fig. 2:**
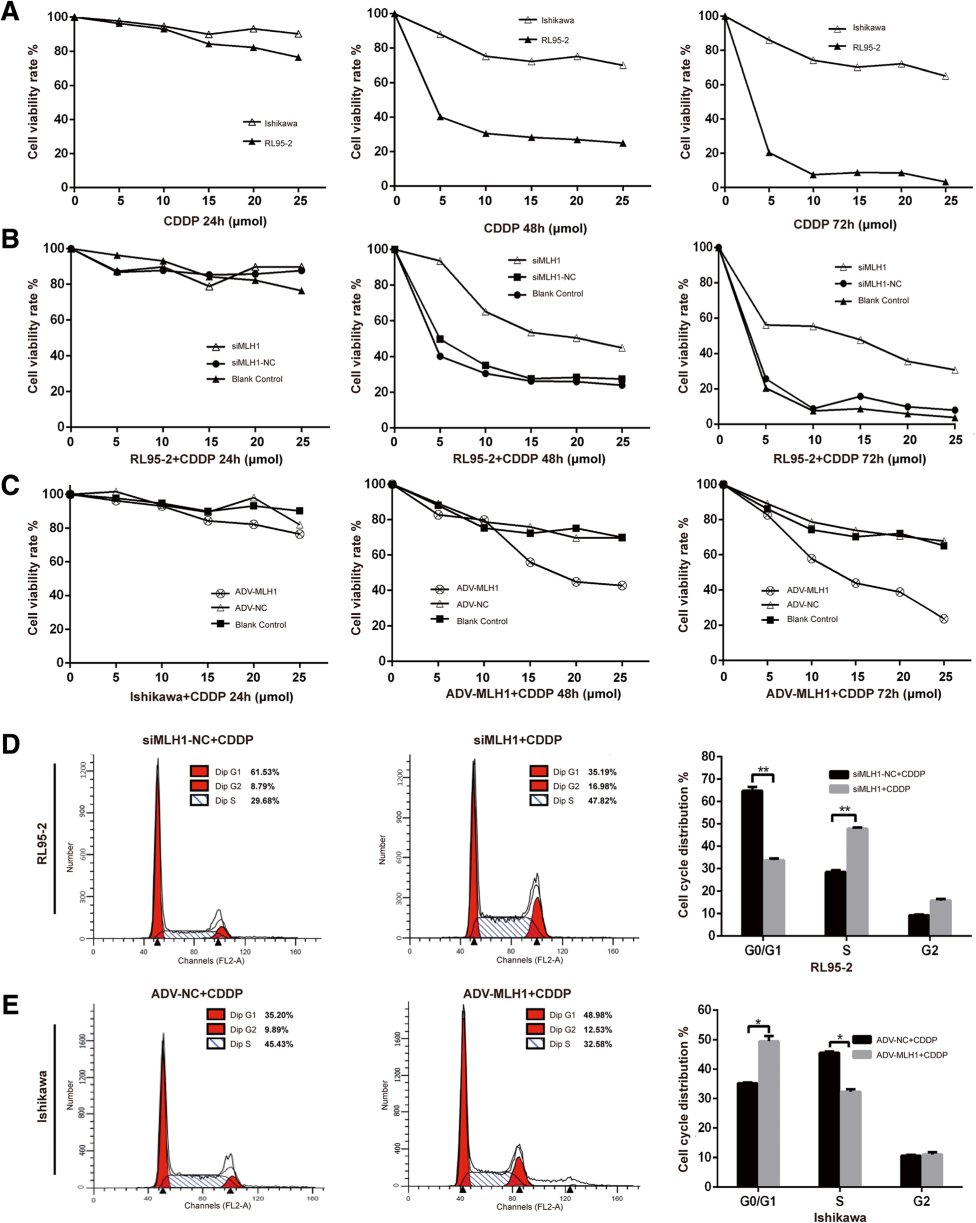
MLH1 affected endometrial carcinoma cell proliferation in response to cisplatin in vitro. **a** RL95–2 and Ishikawa cell growth curves after 24 h, 48 h and 72 h of treatment with different concentrations of cisplatin (0–25 μmol). **b** Viability rates of RL95–2 cells after MLH1 silencing. Cells transfected with siMLH1-NC and normal RL95–2 cells served as controls. **c** Viability rates of Ishikawa cells after MLH1 overexpression. The viability rate of ADV-MLH1/ADV-NC-infected Ishikawa cells was normalized to control cells. Each value represents the mean of at least three independent experiments performed in triplicate (CCK-8). **d** Distribution of the cell cycle in response to 5 μmol cisplatin in MLH1 knockdown RL95–2 cells compared with the control. **e** Distribution of the cell cycle in response to 25 μmol cisplatin in MLH1-overexpressing cells compared with the control

Sensitization to some chemotherapeutic agents is thought to be associated with functional MMR [18, 19]. The different MLH1 expression levels in endometrial carcinoma cells prompted us to examine whether overexpression or knockdown of MLH1 affects cell proliferation in response to cisplatin. Figure 2a presents the results of CCK-8 assays in which MLH1-proficient RL95–2 cells and MLH1-deficient Ishikawa cells were treated with different concentrations of cisplatin (0–25 μmol) for 24, 48 and 72 h. The figure shows that the viability of RL95–2 (MLH1-proficient) cells was considerably reduced compared with that of Ishikawa (MLH1-deficient) cells upon exposure to cisplatin, revealing concentration- and time-dependent suppression (Fig. 2a). Cell viability was significantly increased in siMLH1-transfected RL95–2 cells compared with control cells (Fig. 2b). We also detected significantly decreased tolerance to CDDP in ADV-MLH1-infected Ishikawa cells compared with controls (Fig. 2c). Meanwhile, DMSO (CDDP solvent) alone had no effect on cell viability (data not shown). These results indicated that re-expressing MLH1 increased the sensitivity of Ishikawa cells to cisplatin, and silencing MLH1 decreased RL95–2 cell sensitivity to cisplatin. As illustrated in Fig. 2d and e, a slight increase in the number of cells in G1/G0 (*P* < 0.05) was observed in ADV-MLH1-infected Ishikawa cells, whereas a decrease in the distribution of the G1/G0 peak (*P* < 0.01) and an increase in the percentage of S-phase cells (*P* < 0.01) were observed in MLH1-downregulated cells (RL95–2) compared with the NC group. Altogether, these results indicate that MLH1 affects the sensitivity and cell cycle of endometrial carcinoma cells in response to cisplatin in vitro.

### MLH1 might enhance the cisplatin-induced apoptosis of human endometrial carcinoma cells in vitro

**Fig. 3:**
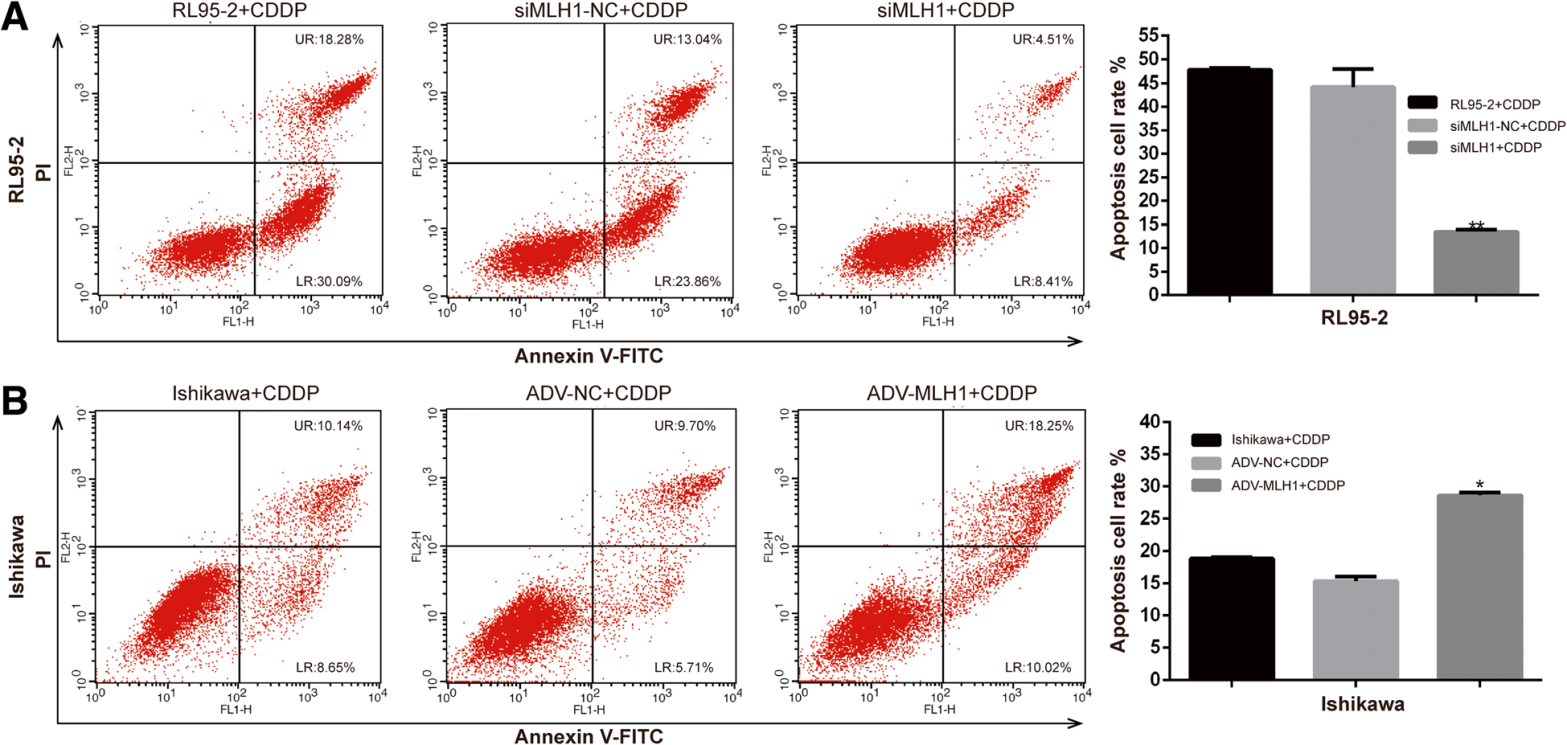
MLH1 enhanced cisplatin-induced apoptosis in endometrial carcinoma cells in vitro. **a** Apoptosis rate of RL95–2 cell 72 h after treatment with 5 μmol cisplatin. **b** Apoptosis rate of Ishikawa cell 72 h after treatment with 25 μmol cisplatin, detected by flow cytometry analysis. UR indicates late apoptosis cells; LR indicates early apoptosis cells

To further characterize the important role of MLH1 in improving the chemosensitivity of human endometrial carcinoma, we assessed the effect of MLH1 on cisplatin-induced apoptosis using flow cytometry. For apoptotic analysis, as illustrated in Fig. 3a and b, the distribution of apoptotic cells was analyzed 72 h after cisplatin treatment. Compared with cells treated with siMLH1/siMLH1-NC, ADV-MLH1/ADV-NC or cisplatin alone, the apoptotic and early apoptotic fractions were increased by 1.51-fold (*P* < 0.05) in ADV-MLH1-infected Ishikawa cells and decreased by 3.54-fold (*P* < 0.01) in siMLH1-transfected RL95–2 cells.

### Cisplatin might activate the MLH1/c-Abl apoptosis signaling pathway in endometrial carcinoma cells

**Fig. 4:**
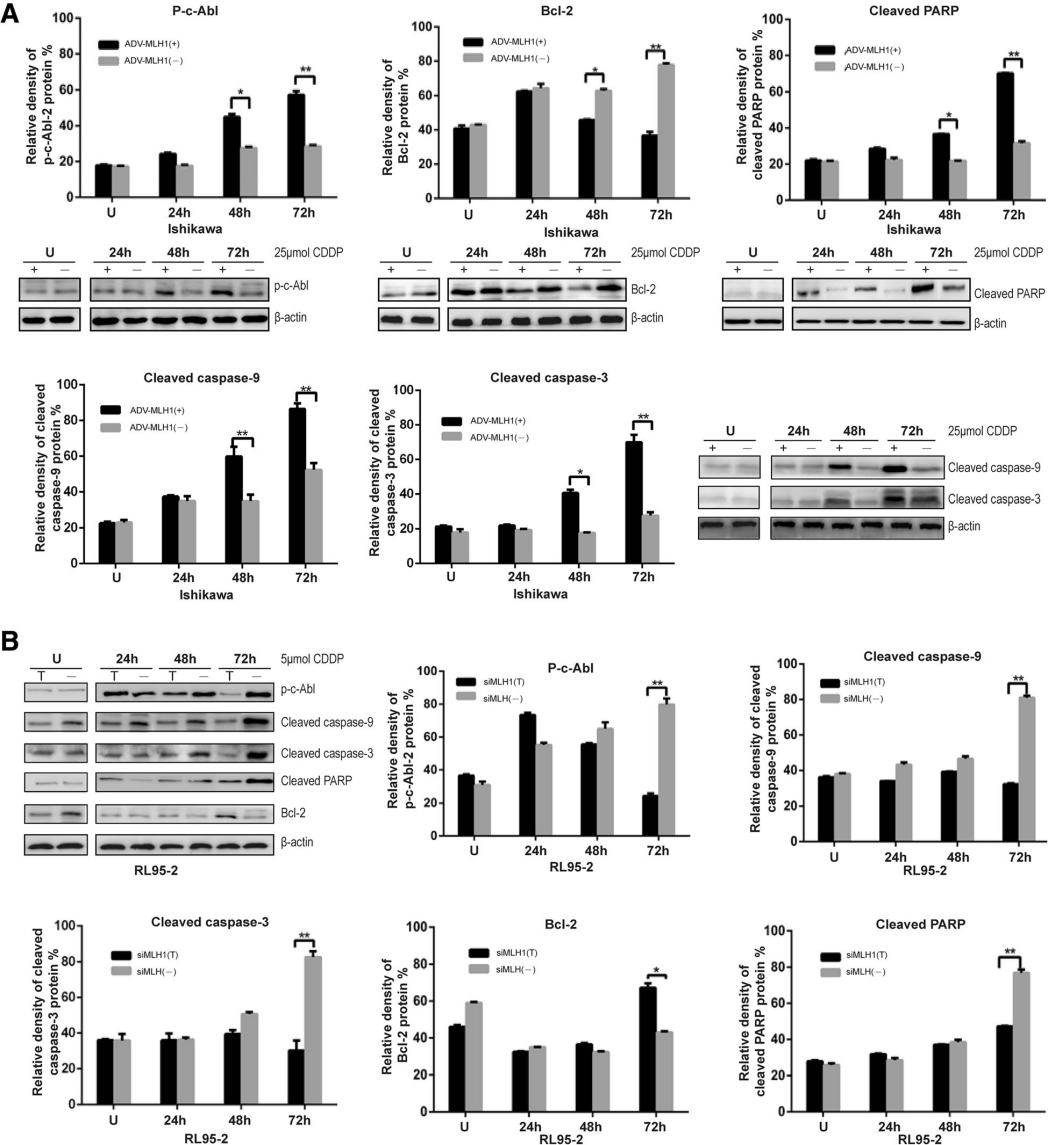
Cisplatin might activate the MLH1/c-Abl apoptosis signaling pathway in ADV-MLH1-infected cells. **a** Immunoblot analysis of p-c-Abl, cleaved caspase-9, cleaved caspase-3, cleaved PARP and Bcl-2 in ADV-MLH1-infected cells (+) 24, 48, and 72 h after exposure to 25 μmol cisplatin compared with same treated MLH1-deficient cells (−). **b** Immunoblot analysis of p-c-Abl, cleaved caspase-9, cleaved caspase-3, cleaved PARP and Bcl-2 in si-MLH1-transfected cells (T) 24, 48 and 72 h after exposure to 5 μmol cisplatin compared with same treated MLH1-proficient RL95–2 cells (−). U: not treated with cisplatin. *, *P* < 0.05; **P < 0.01

Li et al. suggested that c-Abl mediates MLH1-dependent apoptosis in colon cancer cells [20]. Therefore, we examined the potential participation of c-Abl, B-cell lymphoma-2 (bcl-2), caspase-9, caspase-3 and PARP in the cisplatin-induced MLH1/c-Abl apoptosis signaling pathway in endometrial carcinoma cells via Western blot analysis. The phosphorylation of the c-Abl protein was upregulated in Ishikawa cells overexpressing MLH1, demonstrating MLH1 dependence beginning at 48 h and increasing until 72 h after treatment with cisplatin. Quantification of phosphorylated c-Abl processing by densitometry revealed that MLH1-overexpressing Ishikawa cells displayed 1.6- and 2.0-fold increases in c-Abl phosphorylation at 48 and 72 h, respectively, compared with control cells (*P* < 0.01; Fig. 4a). Similar trends were observed for cleaved caspase-9, cleaved caspase-3 and cleaved PARP. Cleaved caspase-3 activity was increased 2.3- and 2.5-fold after the administration of ADV-MLH1 in Ishikawa cells for 48 and 72 h (*P* < 0.01). Compared with control cells, cleaved PARP was increased 1.68-fold at 48 h (*, *P* < 0.05) and 2.2-fold at 72 h (*P* < 0.01) (Fig. 4a). However, when MLH1 expression was downregulated, opposing trends in the levels of these proteins were observed compared with control cells (Fig. 4b).

On the other hand, the expression of the Bcl-2 protein gradually decreased in a time-dependent manner in MLH1-re-expressing Ishikawa cells from 48 h to 72 h after cisplatin treatment (Fig. 4a). When MLH1 expression was downregulated, Bcl-2 exhibited the opposite trend (Fig. 4b).

These results demonstrated that cisplatin might activate the MLH1/c-Abl apoptosis signaling pathway in endometrial carcinoma cells.

### Ishikawa cells infected with ADV-MLH1 display markedly attenuated tumor growth in vivo

**Fig. 5:**
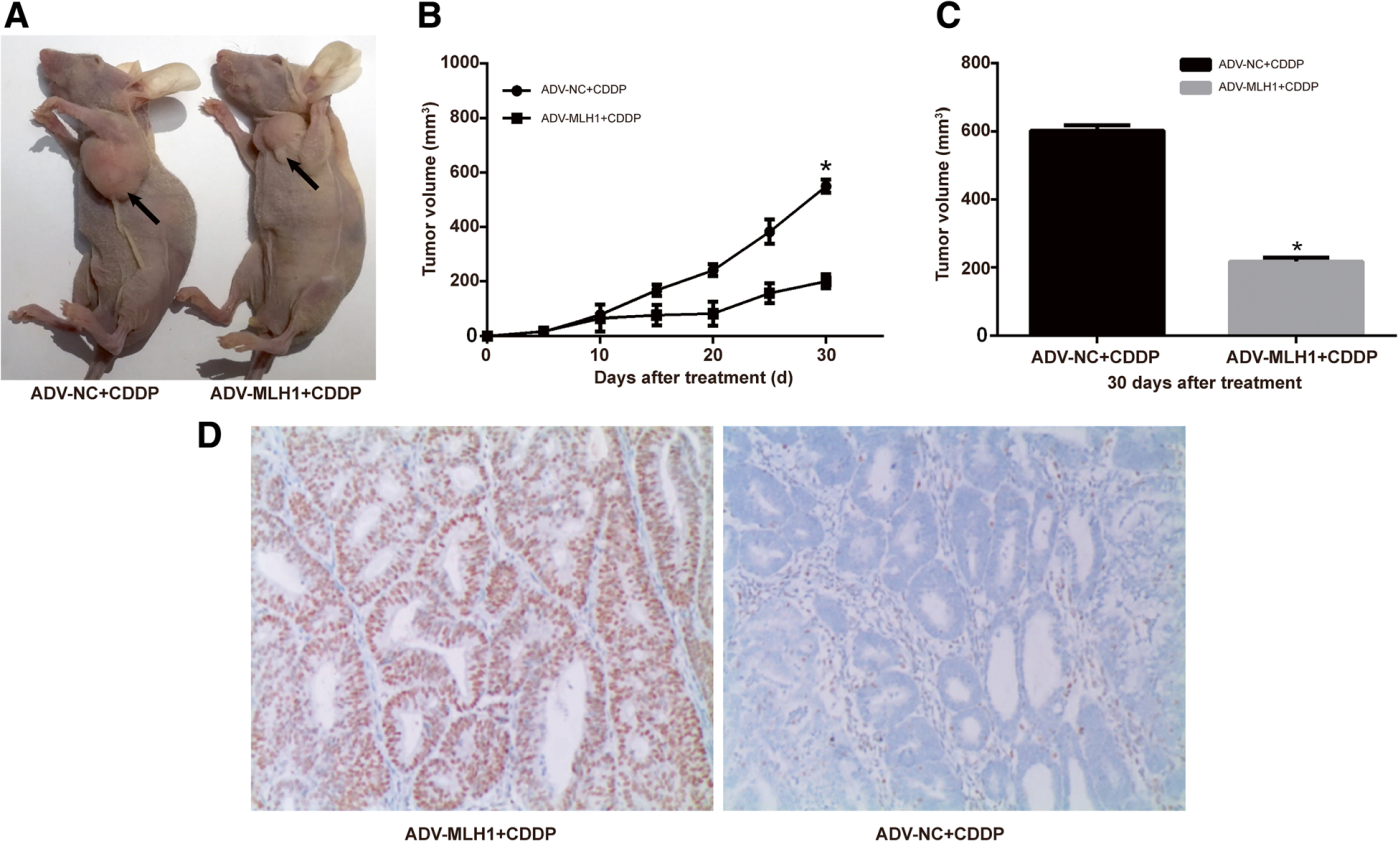
Markedly attenuated tumor growth of Ishikawa cells in vivo in the ADV-MLH1 group. **a** Mice in the ADV-MLH1 + CDDP group developed much smaller tumors than those in the ADV-NC + CDDP group. **b** Growth curve of the tumor size in mice of the ADV-MLH1 group and the ADV-NC group (*n* = 6;*, *P* < 0.05). **c** Comparison of the tumor size in mice of the ADV-MLH1 group (average 207.6 mm^3^) and the ADV-NC group (average 603.0 mm^3^) on day 30. **d** Immunohistochemical analysis of MLH1 protein expression in ADV-MLH1 + CDDP- or ADV-NC + CDDP-treated mouse tumor tissues. Magnification: × 100

We subsequently investigated the effects of MLH1 on tumor growth in response to cisplatin in a mouse xenograft model. The results revealed that the tumors in the ADV-MLH1 group were much smaller than those in the ADV-NC group. On days 25 and 30, mice in the ADV-MLH1 + CDDP group exhibited a significantly reduced tumor size compared with those in the ADV-NC control group (*P* < 0.01, Fig. 5a). On day 30, tumor size in the ADV-NC group increased to an average of 603.0 mm^3^, compared with 207.6 mm^3^ in the ADV-MLH1 group (*P* < 0.05) (Fig. 5b, c). The difference in tumor size between the ADV-MLH1 and ADV-NC control groups was significant. In addition, we performed MLH1 immunohistochemical staining in mouse tumor tissue from each group (ADV-MLH1 + CDDP-treated mice, ADV-NC + CDDP-treated mice) and observed that the ADV-MLH1 + CDDP-treated mouse tumor tissue was MLH1 positive, while the ADV-NC + CDDP-treated mouse group was MLH1 negative (Fig. 5d**)**. These data helped us assess the transduction efficiency in vivo, and the results indicated that ADV-MLH1 might render endometrial carcinoma cells more sensitive to cisplatin and attenuate endometrial tumor growth in vivo.

## Discussion

The human MMR system plays a critical role in maintaining the fidelity of DNA replication. Mismatch recognition is performed by MutSα, which is composed of MSH2 and MSH6, and MutSβ (MSH2-MSH3), whereas the recruitment of downstream MMR proteins is executed by MutLα, which is composed of MLH1-PMS2 [21]. MLH1, which can recognize and repair base-base mismatches and insertion/deletion loops that occur during DNA replication, is the key component of the MMR system. MLH1 also plays an important role in cell apoptosis stimulated by DNA damage [22]. MLH1-deficient cells exhibit resistance when treated with DNA-damaging chemotherapeutic agents [23, 24]. Thus, MLH1 is crucial for maintaining gene integrity in cells and disease prevention. Our study is the first to demonstrate the involvement of MLH1 in cisplatin sensitivity in human endometrial carcinoma cells.

In this study, we detected reduced MLH1 protein and mRNA expression in Ishikawa cells compared with those in RL95–2 cells (Fig. 1a). Because Ishikawa cells are deficient in MLH1 and RL95–2 cells exhibit sufficient MLH1, Ishikawa and RL95–2 cells are ideal cell lines for determining the functional effects of MLH1. By overexpressing or downregulating MLH1 in Ishikawa or RL95–2 cells, respectively, we observed that MLH1 overexpression reduced cell proliferation properties (Fig. 2) and increased cell apoptosis (Fig. 3) in response to cisplatin, indicating that MLH1 plays an important role in the cisplatin sensitivity of human endometrial carcinoma cells. Consistent with our results, MLH1 has been reported to play an important role in cell apoptosis. Ding et al. [25] suggested that in the MMR-deficient ovarian cancer cell line A2780MNU1, MLH1 overexpression significantly reduced cell survival and proliferation, with a high percentage of cells displaying active caspase-3 after drug treatment. Additionally, Ruzov et al. [26] showed that upregulation of MLH1 in HCT116 cells resulted in apoptosis during the onset of gastrulation in embryos. A similar effect was observed in a mouse xenograft model. In the present study, upregulation of ADV-MLH1 led to tumor growth arrest compared with tumors injected with ADV-NC (Fig. 5).

However, the mechanism by which MLH1 induces cell apoptosis in response to cisplatin is not fully understood. The c-Abl protein is a mediator of the MLH1-dependent cellular response to damage [27–29]. c-Abl, which exhibits tyrosine kinase activity, is a member of the nonreceptor tyrosine kinase Abelson superfamily, participating in the regulation of cell apoptosis and transformation, and its activation or mutation presents an important relationship with the malignant transformation of tumor cells. Therefore, we investigated whether c-Abl plays a critical role in cisplatin-induced cell apoptosis. In our study, overexpression of ADV-MLH1 activated the phosphorylation of c-Abl and caused a series of changes in downstream proteins (Fig. 4). Notably, phosphorylated c-Abl was robustly enhanced with prolonged cisplatin treatment. These results suggest that c-Abl phosphorylation moderates the apoptotic effects of cisplatin on endometrial carcinoma cells. Thus, c-Abl phosphorylation is important for apoptotic activation, and Hantschel suggested that phosphorylation occurs at tyrosine sites [30]. Li et al. [20] found that MLH1-overexpressing cells induced cell apoptosis in a p53-independent manner; however, when c-Abl transfectants were knocked down, MLH1-dependent apoptosis was prevented. Kim et al. [28] reported that the MEKK-1/MKK4/JNK/c-Jun pathway was triggered by MLH1 in response to the alkylator N-methyl-N′-nitro-N′-nitrosoguanidine (MNNG). They also found that c-Abl is required for the activation of this signaling cascade by MLH1. These authors observed a functional interacting complex between MLH1 and c-Abl in which MLH1 induced apoptosis, and this effect was dependent on the phosphorylation of c-Abl. Thus, evidence strongly suggests that phosphorylated c-Abl may be correlated with the tumor-suppressive effect of MLH1 in endometrial carcinoma cells. Regarding cisplatin sensitivity, these results further strengthen our understanding of MLH1 and indicate that MLH1 expression levels may be predictors of natural and acquired cisplatin resistance. Furthermore, from a clinical perspective, a decreased MLH1 repair capability is associated with resistance to chemotherapy drugs and a poor cancer prognosis [17].

In this study, we also constructed an adenovirus vector to be used for MLH1 overexpression. ADVs are double-stranded DNA viruses without an envelope. ADVs do not integrate into the host cell genome. Thus, the ADV vectors exhibit high safety and have been increasingly applied in clinical trials related to gene therapy. In our study, intratumoral injection of the ADV-MLH1 vector significantly suppressed tumor growth in vivo. This novel strategy provides a potential tool for gene therapy of endometrial cancer.

Our study had some limitations. Overexpression via ADV typically produces superphysiological levels of a protein that cannot recapitulate the physiological role of the protein. Thus, considerable work should be performed to investigate the mechanisms underlying MLH1 deficiency in endometrial carcinoma. The detailed mechanism is still not completely understood, and other MMR components (MSH2, PMS2, MSH6) may be functionally related to MLH1. All of these topics require further study.

## Conclusions

In conclusion, our study indicated that MLH1 plays an important role in improving the cisplatin sensitization of endometrial carcinoma cells through activation of the MLH1/c-Abl signaling pathway in vitro. Using a mouse xenograft model, we demonstrated that Ishikawa cells infected with ADV-MLH1 displayed markedly attenuated tumor growth in vivo, further supporting a pro-apoptosis role of MLH1 in endometrial carcinoma cells. This newly identified mechanism helps provide a better understanding of carcinogenesis, and the ADV-MLH1 adenovirus vector may serve as a novel potential therapeutic treatment for human endometrial carcinoma.

## Additional files

Additional file 1: **Figure S1.** Adenovirus vector map. (PDF 57 kb)

Additional file 2: **Figure S2.** Atlas of MLH1 Full Length Gene Sequencing. (PDF 821 kb)

Additional file 3: **Figure S3.** Results of MLH1 Full Length Gene Sequencing. (PDF 832 kb)

Additional file 4: **Table S1.** The information about the sequences. (XLS 21 kb)

## Abbreviations

ADV-MLH1: Adenovirus vector-MLH1
ADV-NC: Adenovirus vector-negative control
ARRIVE: Animal Research: Reporting of In Vivo Experiments
BCL-2: B-cell lymphoma-2
CCK-8: Cell counting kit-8
CDDP: Cis-dichlorodiammine platinum
DMSO: Dimethyl sulfoxide
ECL: Enhanced chemiluminescence
EDTA: Ethylenediaminetetraacetic acid
FACS: Fluorescence activated cell sorting
FBS: Fetal bovine serum
M-MLV: Moloney murine leukemia virus
MMR: Mismatch repair
MNNG: N-methyl-N′-nitro-N′-nitrosoguanidine
MOI: Multiplicity of infection
PARP: Poly ADP-ribose polymerase
PCR: Polymerase chain reaction
PI: Propidium iodide
qRT-PCR: Quantitative real time polymerase chain reaction
RPMI: Roswell park memorial institute
SDS: Sodium dodecyl sulfate
