# Supplementary figures and images for: MLH1 enhances the sensitivity of human endometrial carcinoma cells to cisplatin by activating the MLH1/c-Abl apoptosis signaling pathway
Source: BMC Cancer. 2018 Dec 29;18:1294. doi: 10.1186/s12885-018-5218-4 (PMC6311060; doi:10.1186/s12885-018-5218-4)

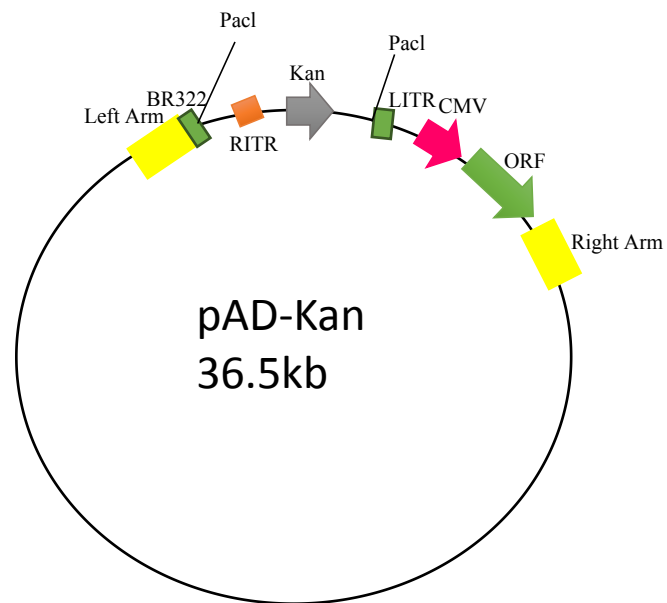

Fig.S1 Adenovirus Vector Map

Supplement: Supplementary file 1 — Figure S1. Adenovirus vector map. (PDF 57 kb) [file 12885_2018_5218_MOESM1_ESM.pdf]

Direction: Native

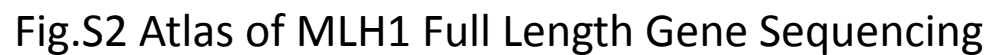

Supplement: Supplementary file 2 — Figure S2. Atlas of MLH1 Full Length Gene Sequencing. (PDF 821 kb) [file 12885_2018_5218_MOESM2_ESM.pdf]
