# Supplementary material for: MLH1 enhances the sensitivity of human endometrial carcinoma cells to cisplatin by activating the MLH1/c-Abl apoptosis signaling pathway
Source: BMC Cancer. 2018 Dec 29;18:1294. doi: 10.1186/s12885-018-5218-4 (PMC6311060; doi:10.1186/s12885-018-5218-4)
Supplement: Supplementary file 3 — Figure S3. Results of MLH1 Full Length Gene Sequencing. (PDF 832 kb) [file 12885_2018_5218_MOESM3_ESM.pdf]

全长基因测序结果

CGCCATGGCTTTCGAGGCTTTGGCCAGCATAAGCCATGTGGCTCATGTTACTATTACAACGAAAACAGCTGA
TGGAAAGTGTGCATACAGAGCAAGTTACTCAGATGGAAAACTGAAAGCCCCTCCTAAACCATGTGCTGGCAA
TCAAGGGACCCAGATCACGGTGGAGGACCTTTTTTACAACATAGCCACGAGGAGAAAAGCTTTAAAAAATCC
AAGTGAAGAATATGGGAAAATTTTGGAAGTTGTTGGCAGGTATTCAGTACACAATGCAGGCATTAGTTTCTC
AGTTAAAAAACAAGGAGAGACAGTAGCTGATGTTAGGACACTACCCAATGCCTCAACCGTGGACAATATTCG
CTCCATCTTTGGAAATGCTGTTAGTCGAGAACTGATAGAAATTGGATGTGAGGATAAAACCCTAGCCTTCAA
AATGAATGGTTACATATCCAATGCAAACTACTCAGTGAAGAAGTGCATCTTCTTACTCTTCATCAACCATCG
TCTGGTAGAATCAACTTCCTTGAGAAAAGCCATAGAAACAGTGTATGCAGCCTATTTGCCCAAAAACACACA
CCCATTCCTGTACCTCAGTTTAGAAATCAGTCCCCAGAATGTGGATGTTAATGTGCACCCCACAAAGCATGA
AGTTCACTTCCTGCACGAGGAGAGCATCCTGGAGCGGGTGCAGCAGCACATCGAGAGCAAGCTCCTGGGCTC
CAATTCCTCCAGGATGTACTTCACCCAGACTTTGCTACCAGGACTTGCTGGCCCCTCTGGGGAGATGGTTAA
ATCCACAACAAGTCTGACCTCGTCTTCTACTTCTGGAAGTAGTGATAAGGTCTATGCCCACCAGATGGTTCG
TACAGATTCCCGGGAACAGAAGCTTGATGCATTTCTGCAGCCTCTGAGCAAACCCCTGTCCAGTCAGCCCCA
GGCCATTGTCACAGAGGATAAGACAGATATTTCTAGTGGCAGGGCTAGGCAGCAAGATGAGGAGATGCTTGA
ACTCCCAGCCCCTGCTGAAGTGGCTGCCAAAAATCAGAGCTTGGAGGGGGATACAACAAAGGGGACTTCAGA
AATGTCAGAGAAGAGAGGACCTACTTCCAGCAACCCCAGAAAGAGACATCGGGAAGATTCTGATGTGGAAAT
GGTGGAAGATGATTCCCGAAAGGAAATGACTGCAGCTTGTACCCCCCGGAGAAGGATCATTAACCTCACTAG
TGTTTTGAGTCTCCAGGAAGAAATTAATGAGCAGGGACATGAGGTTCTCCGGGAGATGTTGCATAACCACTC
CTTCGTGGGCTGTGTGAATCCTCAGTGGGCCTTGGCACAGCATCAAACCAAGTTATACCTTCTCAACACCAC
CAAGCTTAGTGAAGAACTGTTCTACCAGATACTCATTTATGATTTTGCCAATTTTGGTGTTCTCAGGTTATC
GGAGCCAGCACCGCTCTTTGACCTTGCCATGCTTGCCTTAGATAGTCCAGAGAGTGGCTGGACAGAGGAAGA
TGGTCCCAAAGAAGGACTTGCTGAATACATTGTTGAGTTTCTGAAGAAGAAGGCTGAGATGCTTGCAGACTA
TTTCTCTTTGGAAATTGATGAGGAAGGGAACCTGATTGGATTACCCCTTCTGATTGACAACTATGTGCCCCC
TTTGGAGGGACTGCCTATCTTCATTCTTCGACTAGCCACTGAGGTGAATTGGGACGAAGAAAAGGAATGTTT
TGAAAGCCTCAGTAAAGAATGCGCTATGTTCTATTCCATCCGGAAGCAGTACATATCTGAGGAGTCGACCCT
CTCAGGCCAGCAGAGTGAAGTGCCTGGCTCCATTCCAAACTCCTGGAAGTGGACTGTGGAACACATTGTCTA
TAAAGCCTTGCGCTCACACATTCTGCCTCCTAAACATTTCACAGAAGATGGAAATATCCTGCAGCTTGCTAA
CCTGCCTGATCTATACAAAGTCTTTGAGAGGTGTA

Fig.S3 Results of MLH1 Full Length Gene Sequencing
